# Assessing *Posidonia oceanica* Seedling Substrate Preference: An Experimental Determination of Seedling Anchorage Success in Rocky vs. Sandy Substrates

**DOI:** 10.1371/journal.pone.0125321

**Published:** 2015-04-30

**Authors:** Adriana Alagna, Tomás Vega Fernández, Giovanni D Anna, Carlo Magliola, Salvatore Mazzola, Fabio Badalamenti

**Affiliations:** 1 CNR-IAMC, Institute for Coastal Marine Environment, Castellammare del Golfo (TP), Italy; 2 Saipem S.p.A., San Donato Milanese (MI), Italy; 3 CNR-IAMC, Institute for Coastal Marine Environment, Torretta Granitola (TP), Italy; MESC; University of South Alabama, UNITED STATES

## Abstract

In the last decades the growing awareness of the ecological importance of seagrass meadows has prompted increasing efforts to protect existing beds and restore degraded habitats. An in-depth knowledge of factors acting as major drivers of propagule settlement and recruitment is required in order to understand patterns of seagrass colonization and recovery and to inform appropriate management and conservation strategies. In this work *Posidonia oceanica* seedlings were reared for five months in a land-based culture facility under simulated natural hydrodynamic conditions to identify suitable substrates for seedling anchorage. Two main substrate features were investigated: firmness (i.e., sand vs. rock) and complexity (i.e., size of interstitial spaces between rocks). Seedlings were successfully grown in culture tanks, obtaining overall seedling survival of 93%. Anchorage was strongly influenced by substrate firmness and took place only on rocks, where it was as high as 89%. Anchorage occurred through adhesion by sticky root hairs. The minimum force required to dislodge plantlets attached to rocky substrates reached 23.830 N (equivalent to 2.43 kg), which would potentially allow many plantlets to overcome winter storms in the field. The ability of rocky substrates to retain seedlings increased with their complexity. The interstitial spaces between rocks provided appropriate microsites for seedling settlement, as seeds were successfully retained, and a suitable substrate for anchorage was available. In conclusion *P*. *oceanica* juveniles showed a clear-cut preference for hard substrates over the sandy one, due to the root system adhesive properties. In particular, firm and complex substrates allowed for propagule early and strong anchorage, enhancing persistence and establishment probabilities. Seedling substrate preference documented here leads to expect a more successful sexual recruitment on hard bottoms compared with soft ones. This feature could have influenced *P*. *oceanica* patterns of colonization in past and present time.

## Introduction

Seagrass meadows are undergoing worldwide decline due to rapid environmental changes prompted by human activities [1–2]. Over the past few decades, increasing efforts have been made to prevent further losses and to restore degraded habitats through active transplantation, with variable results being obtained depending on the species involved [3–4], methodologies applied and availability of suitable sites [5–6]. Traditional techniques used for this purpose include the manual extraction of plugs, sods or bare-root adult plants from donor populations, followed by transplantation to restoration sites. A major concern regarding the application of these methods at medium and large scales is the potential detrimental effects on donor beds due to the removal of large amounts of biological material [7]. This concern has increased the demand for more ecologically sustainable techniques that support degraded populations without negatively impacting healthy ones. This aim is particularly relevant when working with large, slow-growing species, such as members of the genera *Thalassia* and *Posidonia*.

The so-called donor bed-independent methods address these requirements through the use of seeds and seedlings [8–10], nursery-propagated plants from seeds [11], beach-cast vegetative fragments [12] or the facilitated recruitment of sexual propagules released from adjacent beds through specifically deployed suitable substrates [13–14]. Methods based on the use of sexual propagules also ensure the maintenance of population genetic variability [15] and should be recommended [16–17].

*Posidonia oceanica* L. Delile, the dominant seagrass endemic to the Mediterranean Sea, is undergoing widespread regression [1]. Attempts to restore *P*. *oceanica* meadows using either adult plants or seedlings have produced highly variable results, and no substantial recovery over the long term has been reported by monitoring programs to date [18,3]. However, natural recovery of meadows is rarely documented, particularly in entirely unvegetated areas [but see 19], and may require decades or even centuries [20–21].

*Posidonia oceanica* exhibits particularly slow growth dynamics, the slowest among all seagrasses, resulting in the low recovery rates of its meadows [20–21]. The life history traits of this species cause it to be particularly vulnerable to disturbance, emphasising the need to preserve existing meadows and implement more effective and sustainable restoration methods.

The use of seeds and seedlings can contribute greatly to the development of rehabilitation strategies aimed at minimising impacts to existing beds while maintaining genetic variability. Transitioning to a seed-based strategy requires in-depth knowledge of the processes and factors that influence seedling survival and establishment [8,13,22–23].

*Posidonia oceanica* has no seed dormancy and exhibits highly variable flowering and fruit output, both spatially and temporally. A recent compilation of available data for the Mediterranean indicated that *P*. *oceanica* meadows flower every five years on average, showing events of massive seed production correlated with peaks in the sea surface temperature, suggesting that sexual reproduction might play a more important role in this species than previously thought [24]. Moreover, data from the southern Mediterranean region indicate that flowering is a largely regular phenomenon, although with a highly variable intensity [25–26]. Laboratory and field studies report high germination rates (> 80%, [25,27]), and survival rates in laboratory experiments can exceed 70% after 10 months [28]. Conversely, establishment of seedlings in the field has seldom been described, although high densities of germinated seeds have been observed at highly suitable sites [29,30]. Therefore, the successful initiation of new patches is considered to be rare [24].

Data compiled from the literature suggest that the main mechanism controlling seagrass seedling establishment is not failed germination or low survival *per se*, but the physical dislodgement of plantlets through hydrodynamic forces [13,22–23,30–32]. The capability of seagrasses to withstand mechanical disturbances produced by waves and currents is determined by the strength of their anchorage to the substrate via their roots. Thus, the early life-history phases are expected to be more vulnerable to physical dislodgement than later phases, as young plants lack a well-developed anchorage system [23,33]. Recent field observations show that substrate type is likely the most important driver of successful *P*. *oceanica* seedling establishment, followed by factors related to depth, such as the hydrodynamic intensity and light availability [31,33]. Successful seedling establishment has been observed only on substrates that are sufficiently firm to allow seedling rooting [30–31,34], whereas mobile substrates have been shown to be less suitable [30–31,33]. Anchorage strength appears to be greater where propagules become entangled with topographical structures or are retained within crevices in the substrate (i.e., where there is high complexity at the propagule scale) than on flat surfaces (showing low complexity), as they are less likely to be dislodged by waves and currents while rooting on the former type of substrate [23,35]. In a recent paper the presence of an extensive coverage of root hairs with adhesive properties was documented in *P*. *oceanica* seedlings [36]. Anchorage via sticky root hairs appears to constitute a mechanism for early seedling settlement on vegetated and unvegetated rocky substrates that favour juvenile persistence on hard bottoms compared to mobile ones.

According to this model, the availability of firm and complex substrates may facilitate seedlings establishment and thereby increase the persistence of propagules during early life stages.

The aim of this research was to evaluate whether firm substrates (i.e., hard rock) of varying complexity are more suitable than sand for *P*. *oceanica* seedling establishment. We used seedling anchorage success and seedling growth performance as measures of successful establishment under laboratory conditions simulating moderate hydrodynamic field regimes. Varying levels of complexity were achieved by combining rocks of different sizes. It was predicted that

1. anchorage success would be higher on firm substrates than on mobile ones;
2. on rocky substrates, anchorage success would depend on substrate complexity, i.e., on the size and number of interstitial spaces between rocks; and
3. higher growth performance would be correlated with higher anchorage rates.

## Methods

### Ethics Statement

No specific permit was required for *P*. *oceanica* fruit and seed collection as we used only beach-cast biological material.

### Culture facility and seed collection

**Fig 1:**
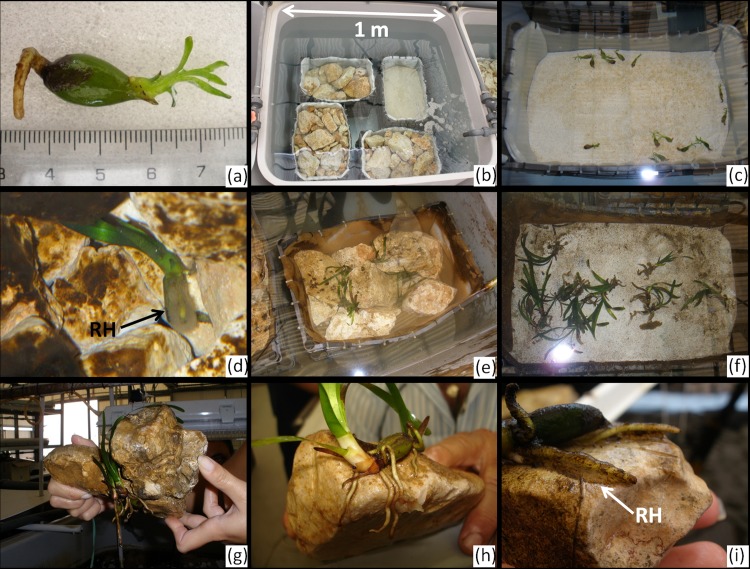
Culture facility and stages of seedling development. (a) *P*. *oceanica* seedling at seeding; (b) one of the six tanks of the culture system (1,000 L), with one box for each level of the experimental treatment: Sand, Rock Low, Rock Medium, Rock High; (c) seedlings immediately after being released on Sand at the beginning of the experiment (June 2010); (d) development of a halo of hairs (RH) on seedling roots; (e) and (f) seedlings growing on rocks and on sand at the end of the experiment (October 2010); (g) and (h) seedlings anchored to rocks and root system development; (i) root hairs (RH) on a rocky substrate covered by the adhesive substance.

The experiments were conducted from June to October of 2010 in a land-based culture facility located at Capo Granitola (Sicily, Italy). The culture system consisted of six small tanks (1 x 1 x 1 m) and one large tank (6 x 1 x 1 m) constructed from fibreglass. The tanks were provided with flow-through natural seawater drawn from a well on the coast. Each tank was fed independently by a pump (30,000 L/minute) and equipped with 6,500 K daylight fluorescent tubes that provided an irradiance of 50 μmoles m^-2^ s^-1^. The tanks were maintained under a 16:8 light:dark photoperiod, and an air pump was used to ensure water oxygenation (Fig 1). The water was sand filtered and UV sterilised before entering the culture system.

The seawater temperature in the tanks ranged from 18.3°C (June) to 20.2°C (October) during the study period.

Beach-cast *P*. *oceanica* fruits and seeds were collected from 15 May to 13 June along the western coast of Sicily (Italy) near Palermo (38°1'N, 13°35'E) and Menfi (37°34'N, 12°52'E). The seeds and fruits were transported to the culture system shortly after collection. All seeds collected or extracted from fruits were rinsed in sterilised seawater and placed in the 6 m^3^ tank prior to the beginning of the experiment.

### Experimental design and statistical analysis

**Fig 2:**
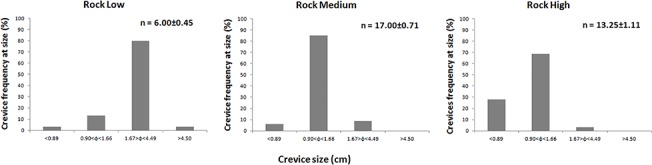
Mean number (n) ± standard error and frequency distribution of crevices according to four size classes at each of the three levels of rocky substrate complexity.

The experiment was designed to test how substrates characterised by different stabilities (sand vs. rock) and complexities (different particle sizes on rocky substrate) affect seedling anchorage and growth under simulated field conditions. Both effects were evaluated using the fixed factor Substrate, which had four levels. One level was composed of carbonatic coarse sand (Sand), and the remaining three consisted of limestone quarry rocks. The three levels of complexity of the rocky substrate were produced by combining different proportions of large cobble (LCo, mean diameter 181.60 ± 0.39 mm, [mean ± SD]), medium cobble (Mco, 145.00 ± 3.10 mm), small cobble (Sco, 105.00 ± 3.10 mm) and coarse gravel (CGr, 55.30 ± 13.60 mm) to achieve the following compositions: 100% [volume] LCo; 76% LCo and 24% CGr; and 31% LCo, 22% Mco, 25% SCo and 22% CGr, yielding low (Rock L), medium (Rock M) and high (Rock H) levels of complexity, respectively. The crevice size frequency distribution was also determined in the three rocky substrates to better characterise their complexity (Fig 2). Crevices were counted and grouped into four dimensional classes according to their width (φ): φ<0.89 cm (I); 0.90 cm <φ<1.66 cm (II); 1.67 cm<φ<4.49 cm (III); and φ>4.50 cm (IV). The threshold values between size classes were chosen according to metrics relevant to seedling-substrate interactions, which included the mean seed width (0.88 ± 0.01 cm), mean seed length (1.65 ± 0.01 cm) and mean seedling length at seeding (4.28 ± 0.21 cm). The results for the chi-square (χ^2^) statistic confirmed significant differences in the frequency distributions of crevice sizes between complexity levels (χ^2^: 89.21 p<0.0010). Sand was considered to have a "null" complexity, as the interstitial spaces between particles were not relevant at the seedling scale.

**Fig 3:**
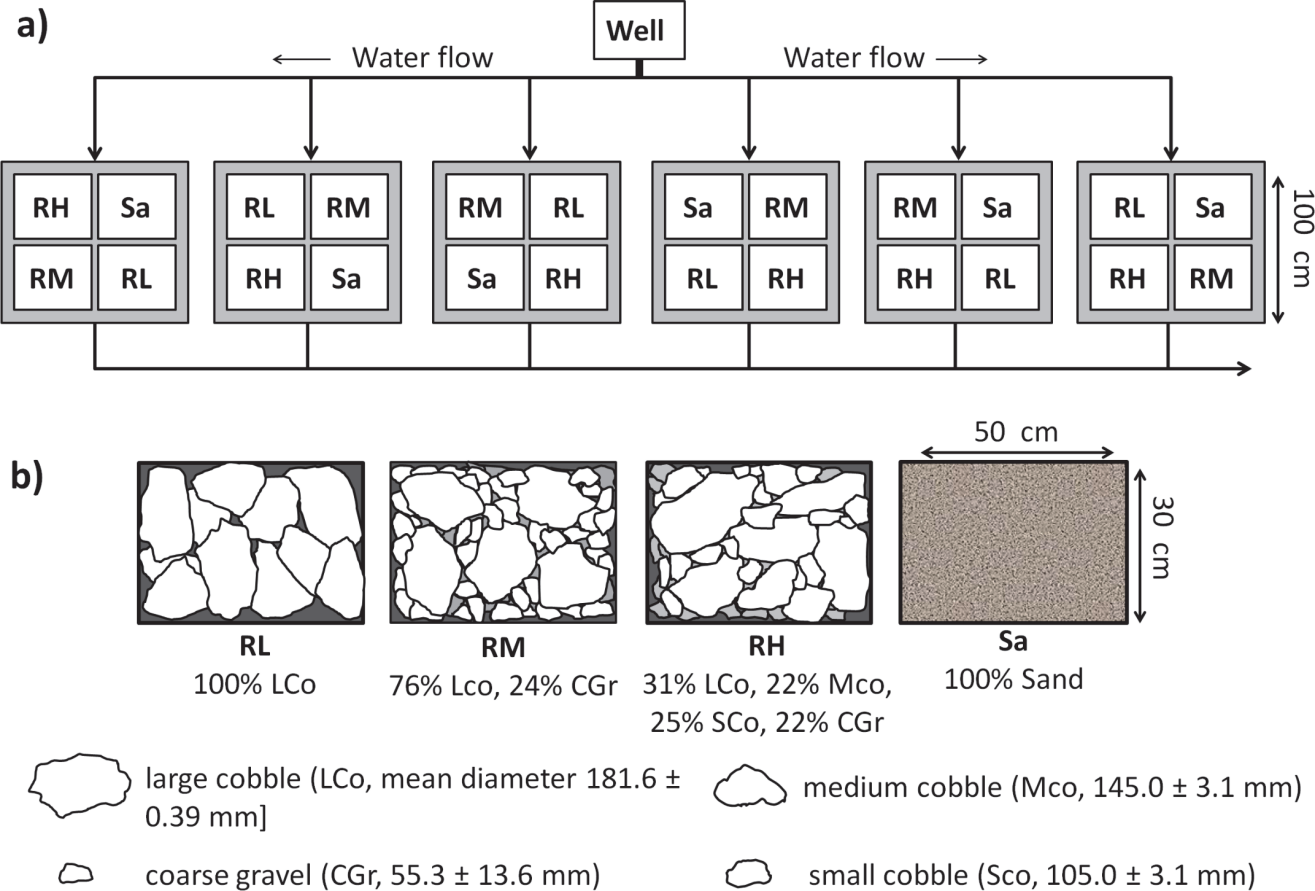
Diagram of experimental system setup: tanks (shadowed squares), boxes (empty squares) and water flow are illustrated. a) RH = Rock High; RM = Rock Medium; RL = Rock Low; Sa = Sand. b) Grain size compositions of the experimental groups contained in each tank.

Six thick plastic-grid boxes measuring 50 x 30 x 27 cm were used for each treatment level. The boxes were open at the top and lined on the bottom to avoid losing small particles. Each box was filled with either one of the three rock assemblages or sand (total number of boxes = 24). The boxes were randomly assigned to one of the six tanks of the culture system such that each tank hosted one box for each treatment level (Figs 1 and 3). The boxes were placed at a depth of 30 cm from the water surface.

A total of 360 homogeneously sized germinating seeds were measured and released above the boxes, at a density of 15 seeds per box. An additional twenty seeds were frozen to assess individual biomass at the beginning of the experiment.

To mimic the seed-substrate interaction under mechanical disturbance by waves, water motion was provided through a vertical wave maker driven by a piston, twice a week. The wave maker was set to produce a near-bottom orbital velocity between 4 and 16 cm s^-1^. This hydrodynamic disturbance is analogous to that experienced by seedlings in the field during their first months of life (see near-bottom orbital velocity values reported by Infantes *et al*. [33] for *P*. *oceanica* meadows at a depth between 12 and 18 m from August to October).

At the end of the experiment, the boxes were disassembled, and seedling survival, anchorage rate (i.e., the percentage of seedlings fixed to the substrate), retention rate (i.e., the percentage of seedlings that remained trapped in the substrate without passing through it) and anchorage strength (i.e., the force needed to detach anchored seedlings from the substrate) were recorded for each box. Growth performance was evaluated *a posteriori* through measurements of seedling morphology and biomass. The obtained morphological measures included the total number of leaves produced (number of leaf sheaths plus the number of standing leaves), total leaf area per seedling, maximum leaf length, maximum leaf width, number of roots, maximum root length, and total root length. The assessed biomass measures consisted of seedling total biomass and the percentages of seed, root and leaf biomass. The seedlings were partitioned into components and individually measured and weighed (precision 0.0001 g) after oven-drying at 70°C for 48 h.

Anchorage strength was measured using a spring scale (precision 5 g), which pulled the plantlet perpendicular to the substrate until detachment [see 36]. The force applied at the point of uprooting was then calculated.

The retention rate was measured as the number of seedlings that did not fall to the bottom of the boxes divided for the total number of seedlings released in each box. This variable was calculated only for those treatment levels composed of rocks.

A one-way ANOVA was performed on survival, anchorage and retention rates including 6 replicates, one for each experimental unit (tank). As anchorage did not occur on Sand, the one-way ANOVA was performed only on three levels: Rock L, Rock M and Rock H.

Anchorage strength and the morphological and biomass variables were analysed following the same experimental design but with the addition of the random factor Tank, orthogonal to the factor Substrate, with six levels. Here, 9 replicates (seedlings) were randomly measured in each experimental unit, with the exception of the variable anchorage strength, for which there were only 6 replicates (seedlings) for each experimental unit. The homogeneity of variances was checked via Cochran’s C tests. Arc sine transformation was needed to achieve homoscedasticity for the following variables: survival, anchorage and retention rates.

The intensity of the hydrodynamic forces that rock-anchored seedlings can potentially withstand in the field before being uprooted was estimated as the maximum near-bottom orbital velocity (*u*, in ms^-1^) from the equation of Koch *et al*. [37]:

$$u={\left(2{\text{F}}_{\text{d}}/{\text{C}}_{\text{d}}\rho \text{A}\right)}^{1/2}$$

where F_d_ is the drag force, measured as the minimum force required to detach seedlings from the substrate; C_d_ is the drag coefficient, offset at 0.1 for *P*. *oceanica* seedlings between 5–9 months of life [33]; ρ is water density, set at 1,025 kg m^-3^; and A is the cross-sectional surface area of the seedling, estimated as the mean leaf area per seedling.

## Results

### Seedling survival, anchorage and retention rates

**Fig 4:**
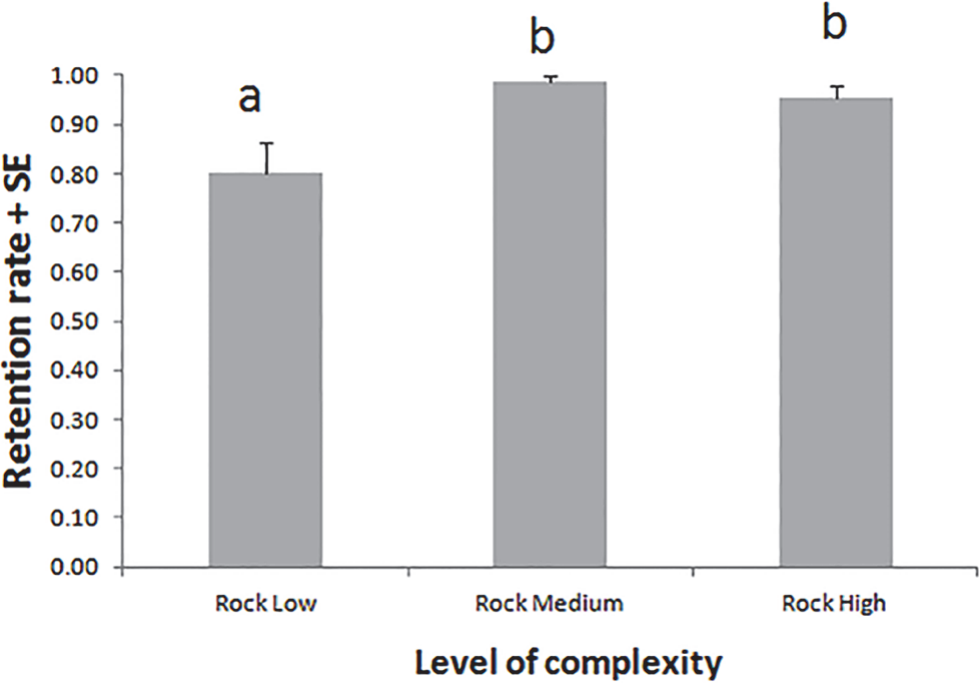
Effect of complexity on the ability of rocky substrates to retain seedlings (retention rate) under a moderate hydrodynamic regime. Letters above standard error bars indicate significant differences between treatment levels.

The survival rate ranged from 0.91 ± 0.04 to 0.95 ± 0.02 (mean ± SE), with an overall mean of 0.93 ± 0.01 being obtained at the end of the experiment. No differences between substrate types and complexity levels were found (F_3, 20_ = 0.28, P = 0.840). No seedlings anchored on sand, and stable settlement on this substrate was never observed during the study period. The progression of seedling anchorage on the bare rocky substrates was observed over the first 3 weeks of culture: the emergence and growth of the root system was coupled with the production of extensive root hairs on the lower part of the seed (hypocotyl region) as well as in the piliferous zone of primary and adventitious roots. Hairs appeared as a halo surrounding the roots and the lower part of the seed. Anchorage occurred via root hair adhesion to the substrate, mediated by a sticky material at the interface between the root hairs and the substrate (Fig 1). The anchorage rates on rocky substrates ranged from 0.84 ± 0.05 (mean ± SE) on Rock L to 0.89 ± 0.04 on Rock H, showing an overall mean value of 0.86 ± 0.01. The anchorage rate did not differ between complexity levels (F_2, 15_ = 0.38, P = 0.688). The ability of the rocky substrates to retain seedlings varied between complexity levels (F_2, 15_ = 6.32, P = 0.010), being significantly higher on both Rock H (0.96 ± 0.02) and Rock M (0.99 ± 0.02) than on Rock L (0.80 ± 0.06), as indicated by the multiple paired comparisons of means (SNK test, Fig 4).

A mean anchorage strength of 2.78 ± 0.37N (mean ± SE) was observed, showing high variability and ranging from 0.10 to 23.83 N. However, ANOVA revealed no significant differences between either complexity levels (F_2, 10_ = 1.39, P = 0.294) or tanks (F_5, 90_ = 1.39, P = 0.235). The estimated maximum near-bottom orbital velocity that a seedling could potentially withstand before being dislodged was highly variable, ranging from 12.82 m s^-1^ to 0.26 m s^-1^, with a mean of 3.22 ± 0.18 m s^-1^.

### Growth performance

**Fig 5:**
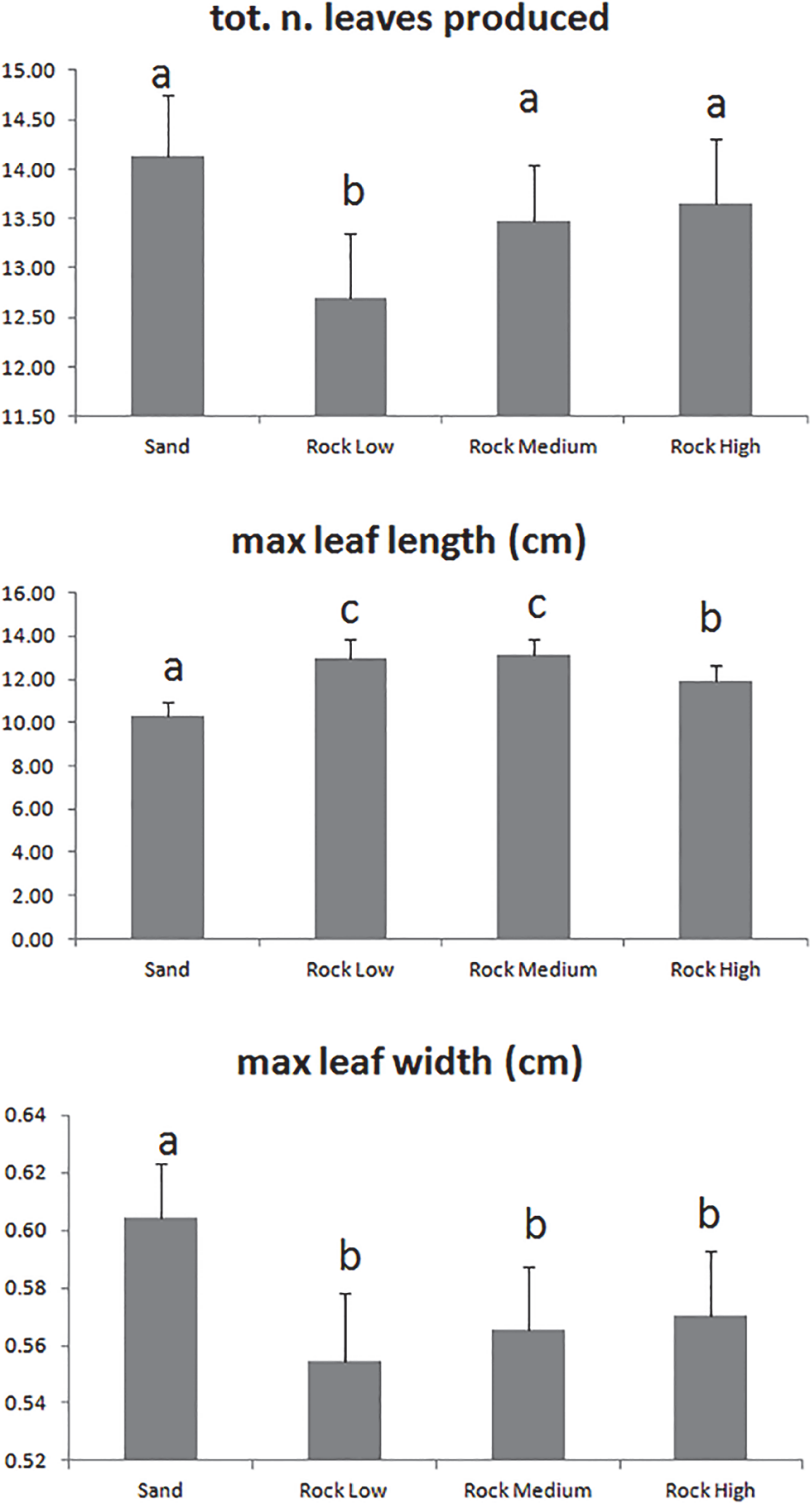
Effect of the Substrate on morphological variables of *P*. *oceanica* seedlings. Mean values + standard errors are shown only for those variables for which a significant effect of the factor Substrate was detected. Letters above standard error bars indicate significant differences between treatment levels.

The total number of leaves produced by seedlings ranged from 12.70 ± 0.66 (mean ± SE) on Rock L to 14.14 ± 0.61 on Sand and was significantly lower on Rock L than on the other substrates (Table 1, Fig 5). No differences in the number of standing leaves were detected between substrates (6.81 ± 0.08). The total leaf area per seedling (28.24 ± 0.49 cm^2^) did not vary across substrates or tanks (Table 1), but the leaves were significantly wider (maximum width 0.60 ± 0.02 cm) and shorter (maximum length 10.27 ± 0.71 cm) on seedlings growing on Sand than those anchored to rocky substrates (maximum width 0.56 ± 0.01 cm; maximum length 12.66 ± 0.49 cm) (Table 1, Fig 5). The maximum leaf length on seedlings grown on rocky substrates was significantly greater on both Rock L (12.98 ± 0.89 cm) and Rock H (13.11 ± 0.78 cm) than on Rock M (11.90 ± 0.78 cm) (Table 1, Fig 5). On average, the seedlings produced 4.05 ± 0.07 roots, with a maximum root length of 5.03 ± 0.12 cm and a total root length of 13.44 ± 0.34 cm being observed. No differences in the morphology of the root system were detected across the substrates or tanks (Table 1).

**Table 1:** Results of ANOVA testing the effect of the Substrate on seedling morphological variables.

Source of variation			*tot n*. *leaves*		*n*. *leaf sheaths*		*max leaf length*		*max leaf width*	
		***df***	***F***	***P***	***F***	***P***	***F***	***P***	***F***	***P***
Substrate	Su	3	8.79	**0.001**	7.63	**0.003**	71.48	**0.001**	10.92	**0.001**
Tank	Ta	5	1.85	0.105	4.08	**0.002**	2.05	0.073	1.57	0.171
Su x Ta		15	0.94	0.518	1.55	0.091	0.34	0.991	0.81	0.662
Residual		192								
Total		215								
			*tot*. *leaf area per seedling*		*n*. *of roots*		*max root length*		*tot root length*	
Source of variation										
		***df***	***F***	***P***	***F***	***P***	***F***	***P***	***F***	***P***
Substrate	Su	3	2.56	0.094	2.40	0.111	1.71	0.208	3.72	**0.035**
Tank	Ta	5	1.13	0.348	1.30	0.267	1.59	0.166	0.64	0.672
Su x Ta		15	0.38	0.982	2.03	**0.015**	2.11	**0.011**	1.89	**0.027**
Residual		192								
Total		215								

Numbers in bold indicate significant effects.

**Fig 6:**
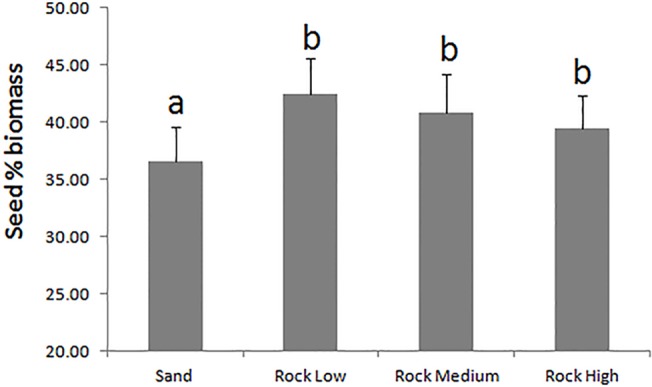
Effect of the Substrate on the seed % biomass in *P*. *oceanica* seedlings. Letters above standard error bars indicate significant differences between treatment levels.

**Fig 7:**
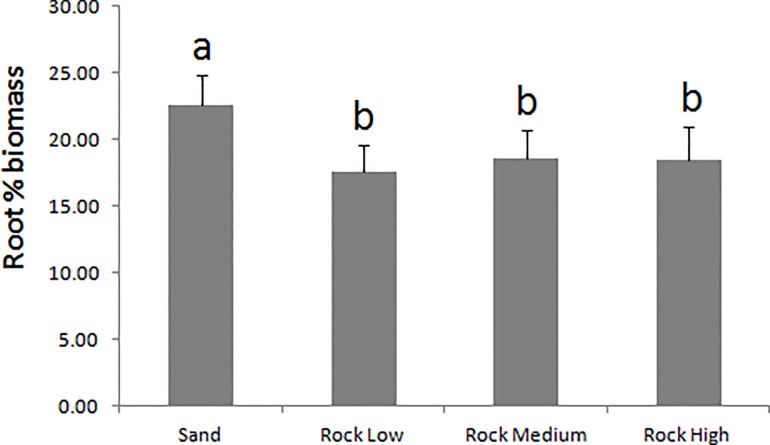
Effect of the Substrate on the root % biomass in *P*. *oceanica* seedlings. Letters above standard error bars indicate significant differences between treatment levels.

Overall seedling biomass increased from a mean of 0.28 ± 0.02 g at the beginning of the experiment to a mean of 0.31 ± 0.01 g after 5 months. Seedling total biomass did not vary between substrates, whereas resource allocation did (Table 2). On Sand, the seedlings showed a significantly lower seed % biomass (36.58 ± 3.02) and higher root % biomass (22.60 ± 2.25) than on the rocky substrates (40.90 ± 0.61 seed % biomass and 18.21 ± 0.14 root % biomass) (Table 2, Figs 6 and 7). No differences were found in the leaf % biomass (40.87 ± 0.40) between the substrates or tanks (Table 2).

**Table 2:** Results of ANOVA testing the effect of the Substrate on seedling biomass.

Source of variation			*tot biomass*		*seed % biomass*		*root % biomass*		*leaf % biomass*	

		*df*	*F*	*P*	*F*	*P*	*F*	*P*	*F*	*P*
Substrate	Su	3	0.26	0.850	5.97	**0.001**	7.39	**>0.001**	1.68	0.173
Tank	Ta	5	1.26	0.285	2.27	0.050	2.03	0.076	1.51	0.189
Su x Ta		15	0.71	0.770	1.14	0.320	1.23	0.253	0.63	0.850
Residual		192								
Total		215								

Numbers in bold indicate significant effects.

## Discussion

*Posidonia oceanica* seedlings were successfully grown from beach-cast fruits and seeds on a large scale in a land-based culture facility that provided a controlled experimental system for investigating seedling substrate requirements. Overall, the seedling survival rate was high, but seedlings settled only on rocky substrates. Settlement on bare rocks was highly successful, with the obtained anchorage probabilities reaching 89%. Anchorage was strongly dependent on the physical stability of the substrate, as settlement occurred only on firm substrate (i.e. limestone rock), and not on sand. This finding is in agreement with previous field observations of seedling persistence and successful establishment on firm substrates, such as rocks covered by algae and dead matte, and of failed persistence on unconsolidated, mobile substrates, such as gravel or sand [30,31,34,38]. The failed or limited establishment of seagrass seedlings on mobile substrates has been attributed to the physical instability of such substrates, thus preventing anchorage, as well as to the abrasive action of the particles on young plantlets [13,31–32,39].

The minimum force needed to dislodge seedlings from rocky substrates was comparable to that recorded *in situ* for seedlings growing on volcanic cobbles [36]. Additionally, the uprooting force recorded in the present study is more than one order of magnitude higher than that determined for *Amphibolis antartica* seedlings entangled in dead seagrass matte and more than two orders of magnitude higher than the value found for the same species on sand by Rivers *et al*. [23]. Badalamenti *et al*. [36] recently documented the presence of extensive root hairs with adhesive properties in *P*. *oceanica* seedlings, which have an anchoring function. The present study confirmed this mechanism more formally with a manipulative approach, as seedling anchorage was observed to take place through the production of root hairs and their subsequent adhesion to the bare rocky substrate via a sticky substance. This mechanism effectively anchors the seedling during its early life stages, prior to the full development of its root system. This finding explains how *P*. *oceanica* is able to colonise hard bottoms through sexual propagules and accounts for the preference of seedlings for hard and firm substrates over soft and mobile ones, as observed in the present work and in accordance with field studies [30–31].

In our experiment, the complexity levels of the rocky substrates did not affect the probability of seedling anchorage or anchorage strength. However, the complexity significantly influenced the ability of the rocky matrix to retain seedlings, as the retention rate increased with complexity, i.e., with decreasing interstitial space size. Processes affecting seedling dispersion and settlement onto a substrate have a stochastic component that makes predictions of the exact outcome of this interaction difficult. However, the medium- and high-complexity levels tested here (Rock M, Rock H) provided a sufficient number of interstitial spaces of a suitable size to allow seeds to enter and remain trapped within the rock matrix, increasing the chances of settlement. The lower retention rate observed on Rock L must be due to the presence of crevices larger than the mean seedling length in this level of the treatment (Fig 2).

In our experiment we simulated the seed- and seedling-substrate interaction under a moderate level of hydrodynamic disturbance. The hydrodynamic forces applied were similar to those found on the average mid-summer and early-autumn field conditions [33]. However, simulating year-round seasonal patterns, including more benign early-summer as well as more harsh late-autumn conditions, would have allowed for a more complete evaluation of seedling anchorage success in the field. It cannot be excluded that in particularly good weather conditions or in sheltered bays (i.e. near bottom orbital velocities < 5 cm s^-1^, [33]), seedlings could produce root hairs and anchor, at least to some extent, even in sediment. Consequently, when autumn harsher conditions arrive, plantlets may have achieved a higher resistance to dislodgement and may recruit. An example of this potential success has been reported recently for a sheltered site along the Tuscanian coast [40]. Unfortunately our work lacked to test for these different environmental conditions.

In this work *P*. *oceanica* seedling substrate preference was assessed contrasting rocks with different levels of complexity against sand. Regretfully, in our experiment we lacked to test for seedling anchorage ability on *P*. *oceanica* dead matte, possibly the most preferred substrate on which undertake restoration actions. Indeed, transplantation trials of *P*. *oceanica* sexual propagules on dead matte have been proved to be successful in fields studies [31, 34] showing that this substrate could be suitable for restoration initiatives. Further experiments should be aimed at assessing the suitability of dead matte as a preferred substrate for seed recruitment, testing whether or not sticky root hairs can play a role by increasing the adhesion strength also in this substrate.

The estimated maximum near-bottom orbital velocity (*u*) provides an indication of the hydrodynamic forces that seedlings anchored to rocks might tolerate in the field before being uprooted. The maximum near-bottom orbital velocities reported in literature at the shallow sites where *P*. *oceanica* occurs range from less than 5 cm s^-1^ in August to 39 cm s^-1^ during winter storms [33]. According to the present study, a seedling anchored to rock might withstand near-bottom orbital velocities one order of magnitude higher than thosje recorded in the field. Nevertheless, there is high variability in seedling anchorage strength and, hence, in the *u* values tolerated by plantlets. We estimated that peak winter storms would uproot 16.7% of the seedlings anchored to rock, while the remaining 83.3% would persist through the winter season. These findings corroborate numerous observations of *P*. *oceanica* seedlings successfully recruited on rocky shores [30–31,41–42]. High anchorage strength on rocky substrates would account for the differences in seedling persistence and survival between the present study and that of Infantes *et al*. [33]; in the latter study, the majority of *P*. *oceanica* seedlings were swept away during the first autumn storms, leading to complete seedling loss before the end of the winter.

The observed differences in seedling morphology and biomass allocation between treatment groups can be explained by plantlet plasticity in response to external stimuli. On the rocky substrates, the seedlings developed within interstitial spaces, where the light intensity was lower than on the open bottom. We propose that the seedlings responded to the lower light availability by developing longer, thinner leaves. Conversely, the seedlings were more exposed to light on the flat sand and produced shorter, larger leaves. Furthermore, plantlets that were not anchored to the sandy substrate allocated more resources to developing the root system, most likely to find anchorage, as shown by the higher root % biomass and lower seed % biomass relative to seedlings growing on rock.

The results of the present study and previous field and laboratory observations [36] support the following model of *P*. *oceanica* seedling establishment. After dehiscence of the floating fruit, the seed falls to the bottom, where it is moved about by waves and currents. In the same time leaf and root primordia begin to develop, and the lower part of the seed (hypocotyl region), the primary roots and adventitious roots begin to produce extensive root hairs that stick to whatever they come into contact with. Prolonged and undisturbed contact between the root hairs and the substrate is required for adhesion. When the seedling comes into contact with a soft substrate, sand grains adhere to the root hairs. Sand grains increase seedling weight, but in the presence of a hydrodynamic regime as the one we simulated, this will not be sufficient to permit the seedling to remain and take root. Waves and currents move the propagule back and forth with the sand grains attached to its roots, hampering settlement and ultimately causing plantlet scouring and death. In contrast, when a seedling encounters a hard substrate (vegetated or unvegetated), the root hairs tend to adhere to it. The topographical complexity of the bottom and the presence of crevices of an appropriate size provide protection from physical disturbances exerted by hydrodynamic forces, favouring prolonged contact between the root hairs and the substrate and therefore adhesion. This process enables firm anchorage to solid substrates to occur during initial seedling establishment, permitting the seedling to remain in place while the root system has yet to develop. After adhesion, the root system develops, providing further mechanical anchorage and entanglement to the substrate. Anchorage strength might further increase with bottom rugosity during this last phase.

The same pattern is observed when *P*. *oceanica* colonisation takes place through vegetative fragments, as shown by a case study in the Capo Feto meadow (western Sicily), where successful and persistent natural recovery occurred on limestone rubble [19, 43]. In contrast, successful establishment of both sexual and vegetative propagules on soft bottoms has only been reported twice to our knowledge, in sheltered, enclosed bays [29, 40].

Taken together, these observations reveal a preference of *P*. *oceanica* during its early life-history phases for firm and complex substrates over mobile ones. Such a preference has far-reaching ecological implications, as *P*. *oceanica*, which has historically been described as a species growing on sandy bottoms, appears to require firm substrates with small-scale complexity to initiate colonization. Substrate preference of *P*. *oceanica* seedlings documented here leads to predict a more successful sexual recruitment on hard bottoms compared with soft ones. This feature could have influenced *P*. *oceanica* patterns of colonization and recovery in past and present time.

In conclusion this study sheds light on factors and mechanisms that control *P*. *oceanica* seedling settlement and recruitment, showing that hard substrates have higher potential compared to soft ones to provide suitable microsites for *P*. *oceanica* seedling settlement. These findings contribute to the development of a comprehensive knowledge of the habitat requirements of the species, in particular that of early life history phases.
